# Draft genome sequence of *Devosia* sp. WQ 349k1, isolated from the feces of *Bos taurus*

**DOI:** 10.1128/mra.00960-24

**Published:** 2025-06-17

**Authors:** Qiong Wang, An-hua Shi, Xiao-song Zhu

**Affiliations:** 1 Center for Pharmaceutical Sciences, Faculty of Life Science and Technology, Kunming University of Science and Technology, Chenggong Campus 47910, Kunming, China; 2 Education Department of Yunnan, Key Laboratory of Microcosmic Syndrome Differentiation, Yunnan University of Chinese Medicine 177554 , Kunming, China; 3 TCM Nutritional Immunology, Cultivating Discipline, Yunnan University of Chinese Medicine 177554 , Kunming, China; University of Maryland School of Medicine, Baltimore, Maryland, USA

**Keywords:** draft genome, *Devosia* sp., strain WQ 349k1

## Abstract

Here, we report the draft genome sequence of a potential novel strain *Devosia* sp. WQ 349k1, isolated from the feces of *Bos taurus. Devosia* sp. WQ 349k1 is a deoxynivalenol-degrading bacterial species. This genome information will help further study its molecular mechanism of detoxification.

## ANNOUNCEMENT

Strains from *Devosia* spp. were characterized for their biodetoxification of deoxynivalenol (1, 2). To investigate its molecular mechanism, the draft genome of an aerobic *Devosia* sp. WQ 349k1 strain was sequenced. Strain WQ 349k1 was isolated from the feces of *Bos taurus* foraging on the Baima Snow Mountain (27°39'N, 99°21'E; elevation, 3000 m), Yunnan province, China. The collection, preservation, and preprocessing of samples were conducted as described by Wang et al. (3). The fresh feces were collected immediately after defecation with sterilized tubes. Samples were temporarily stored in 4°C portable incubators. After a series of gradient dilutions with 0.1% Na_4_P_2_O_7_, samples were evenly spread on brain heart infusion (BHI) agar plates (Hopebio, China) and cultured at 30°C for 3 to 7 days. Single colonies were selected and purified. The pure culture was preserved at 4°C in slants and at −80°C with 20% (v/v) glycerol.

Genomic DNA was isolated using the sodium dodecyl sulfate (SDS) method from cells cultured overnight in BHI agar at 30°C (4). The 16S rRNA gene was amplified with primers 27F (5′-AGAGTT TGATCCTGGCTCAG-3′) and 1492R (5′-GGTTACCTTGTTACGACTT-3′) as described by Han et al. (5). Genomic DNA was randomly broken into 350 bp fragments. NEBNext Ultra DNA Library Prep Kit for Illumina (NEB, USA) was used to construct the library. DNA fragments were processed by terminal repair, adding A-tail, sequencing splice, purification, and PCR amplification. Qubit 2.0 was used for preliminary quantification of the library. Agilent 2100 and qPCR were used to detect the quality of the inserted fragments and the effective concentration of the library, respectively. The genome was sequenced with the Illumina NovaSeq PE150 platform. Readfq (version 10) was used to filter the raw data, and the filtered Clean Data were assembled by SOAP denovo (version 2.04) (6, 7), SPAdes (8), ABySS (9), and integrated by CISA (10). Gapclose (version 1.12) was used to optimize and obtain the final assembly results. The genome was annotated through the NCBI Prokaryotic Genome Annotation Pipeline (11). GeneMarkS (version 4.17) was used to predict the encoded genes (12). For all of these software tools, default parameters were used unless otherwise stated.

Phylogenetic analysis based on 16S rRNA gene sequence revealed that the isolate belongs to the genus *Devosia*, sharing 98.01% similarity with its most closely related strain *Devosia epidermidihirudinis* E 84^T^. The total number of Illumina reads and sequence depth were 927 Mb and 250×, respectively. The draft genome of strain WQ 349k1 was composed of 15 contigs with a maximum length of 1,097,122 bp and a minimum length of 869 bp, and the total genome size was 3.7 Mb with a G+C content of 57.43%, N50 length of 358,202 bp, and N90 length of 201,103 bp. The numbers of coding genes and tRNA were 3,542 and 66, respectively.

## Data Availability

The GenBank accession number for the 16S rRNA gene sequence of strain WQ 349k1 is PV017750. The Whole Genome Shotgun project has been deposited at DDBJ/ENA/GenBank under accession number NZ_JAENGO000000000. The raw sequencing reads have been deposited to the SRA database under accession number PRJNA1215679.
